# Spectral Profiling (Fourier Transform Infrared Spectroscopy) and Machine Learning for the Recognition of Milk from Different Bovine Breeds

**DOI:** 10.3390/ani14091271

**Published:** 2024-04-24

**Authors:** Anna Antonella Spina, Carlotta Ceniti, Rosario De Fazio, Francesca Oppedisano, Ernesto Palma, Enrico Gugliandolo, Rosalia Crupi, Sayed Haidar Abbas Raza, Domenico Britti, Cristian Piras, Valeria Maria Morittu

**Affiliations:** 1 Department of Health Sciences, “Magna Græcia University” of Catanzaro, Campus Universitario “Salvatore Venuta” Viale Europa, 88100 Catanzaro, Italy; aa.spina@unicz.it (A.A.S.); ceniti@unicz.it (C.C.); rosario.defazio@studenti.unicz.it (R.D.F.); palma@unicz.it (E.P.); britti@unicz.it (D.B.); 2 Department of Health Sciences, Institute of Research for Food Safety & Health (IRC-FSH), “Magna Græcia University” of Catanzaro, Campus Universitario “Salvatore Venuta” Viale Europa, 88100 Catanzaro, Italy; foppedisano@unicz.it; 3 Interdepartmental Center Veterinary Service for Human and Animal Health, “Magna Græcia University” of Catanzaro, CISVetSUA, Campus Universitario “Salvatore Venuta” Viale Europa, 88100 Catanzaro, Italy; morittu@unicz.it; 4 Nutramed S.c.a.r.l., Complesso Ninì Barbieri, Roccelletta di Borgia, 88021 Catanzaro, Italy; 5 Department of Veterinary Science, University of Messina, 98166 Messina, Italy; egugliandolo@unime.it (E.G.); rcrupi@unime.it (R.C.); 6 Guangdong Provincial Key Laboratory of Food Quality and Safety, Nation-Local Joint Engineering Research Center for Machining and Safety of Livestock and Poultry Products, South China Agricultural University, Guangzhou 510642, China; haiderraza110@scau.edu.cn; 7 Department of Medical and Surgical Sciences, “Magna Græcia University” of Catanzaro, Campus Universitario “Salvatore Venuta” Viale Europa, 88100 Catanzaro, Italy

**Keywords:** cow milk, Podolica cow milk, (FTIR) spectral profiling, machine learning, multivariate analysis, traceability

## Abstract

**Simple Summary:**

Fourier Transform Infrared Spectroscopy (FTIR) is a rapid, cost-effective, and routinely used tool for milk analysis that can be easily applied to the classification of valuable dairy products such as Podolica milk. In the work herein presented, we applied machine learning to rapidly classify the FTIR datasets of milk from different bovine breeds. We were able to successfully recognize non-Podolica milk with 86% sensitivity and 100% specificity, demonstrating that the combination of these tools might be used in the future for the rapid classification of milk from different bovine breeds.

**Abstract:**

The Podolica cattle breed is widespread in southern Italy, and its productivity is characterized by low yields and an extraordinary quality of milk and meats. Most of the milk produced is transformed into “Caciocavallo Podolico” cheese, which is made with 100% Podolica milk. Fourier Transform Infrared Spectroscopy (FTIR) is the technique that, in this research work, was applied together with machine learning to discriminate 100% Podolica milk from contamination of other Calabrian cattle breeds. The analysis on the test set produced a misclassification percentage of 6.7%. Among the 15 non-Podolica samples in the test set, 2 were misclassified and recognized as Podolica milk even though the milk was from other species. The correct classification rate improved to 100% when the same method was applied to the recognition of Podolica and Pezzata Rossa milk produced by the same farm. Furthermore, this technique was tested for the recognition of Podolica milk mixed with milk from other bovine species. The multivariate model and the respective confusion matrices obtained showed that all the 14 Podolica samples (test set) mixed with 40% non-Podolica milk were correctly classified. In addition, Pezzata Rossa milk produced by the same farm was detected as a contaminant in Podolica milk from the same farm down to concentrations as little as 5% with a 100% correct classification rate in the test set. The method described yielded higher accuracy values when applied to the discrimination of milks from different breeds belonging to the same farm. One of the reasons for this phenomenon could be linked to the elimination of the environmental variable. However, the results obtained in this work demonstrate the possibility of using FTIR to discriminate between milks from different breeds.

## 1. Introduction

The Podolica cattle breed is widespread in southern Italy. It is usually bred in a wild or semi-wild state in the mountains, with a life expectancy of about 15 years [1]. In general, Podolica cattle have ancient European origins tracing back to *Bos primigenius*, and they are characterized by a grey coat and long horns [2,3]. The productivity of Podolica cows is characterized by low yields and an extraordinary quality of milk, as well as by valuable and appreciated meats. Most of the milk produced is used in the production of “Caciocavallo Podolico” cheese that is made with 100% Podolica milk [3]. The valorization of this product requires cheap and easily applicable tools for the rapid recognition of this milk or of mixtures of this product with milk of other breeds. In fact, milk adulteration of a more valuable breed with the milk of different breeds or species with lower economical value is frequent [4]. The consequences of this practice may result in the milk losing authenticity and quality while also causing the onset of problems related to allergies and changes in the content of bioactive molecules capable of conferring protection from microbial infections and inflammation [4,5]. Being able to easily and cost-effectively protect this dairy product would produce a positive impact not only in terms of animal welfare, but also in terms of the sustainability and rural recovery of degraded areas.

In order to guarantee the authenticity of milk to both producers and consumers, various very expensive and sophisticated analytical methods have been applied, such as chromatographic techniques combined with mass spectrometry [5]. Today, however, there is a reliable, simple, and rather quick method to discriminate between authentic and adulterated milk. This technique associates vibrational spectroscopy with chemometrics. In particular, the authenticity of milk and dairy products is obtained by studying the interaction of the samples with electromagnetic radiation in the infrared region and processing the data obtained through mathematical and statistical techniques that allow for the construction of recognition and discrimination models [4,5,6,7,8,9,10]. This technique is Fourier Transform Infrared Spectroscopy (FTIR) [11,12,13], which is applied to evaluate, for example, the eventual changes in milk composition caused by changes in the welfare status of dairy cows following changes in housing conditions [14,15] or to classify and discriminate between different milk sources (such as cow, sheep, and water buffalo milk) in order to quantitatively, quickly, and very accurately detect sample adulteration [16]. This technique was also applied to identify adulterations to samples of cow and buffalo milk after the addition of soy milk [17].

The study of milk from different bovine breeds was previously conducted by combining the results of infrared spectroscopy and liquid chromatography. The obtained results shed light about the detailed milk protein profile of different cattle breeds [18] that could offer significant advantages to the dairy sector, offering fresh resources for improving milk payment systems based on recognition and the design of breeding programs.

In this study we tried to implement a different method that uses the datasets produced by the FTIR analysis (subjected to multivariate analysis) of different farms of four different bovine breeds, including Podolica. The datasets were collected and subjected to multivariate analysis and machine learning for rapid recognition of the type of samples and of the different experimental mixtures. The scope of this work was to create FTIR-based multivariate models applicable for the correct recognition of the analyzed samples.

## 2. Materials and Methods

### 2.1. Samples Collection

The study enrolled bulk milk samples from three different Podolica farms and four different farms of other bovine breeds (Frisona, Pezzata Rossa, and Bruna Alpina). Two different samples (bulk tank, average of the entire herd) were collected at two different timepoints (interval of at least one week) from each farm to enhance variability.

The Podolica heads in lactation were 50, 40, and 30 for the 3 different farms (120 in total). In these farms, all the samplings were carried out through the use of mobile milking machines.

All the Podolica herds sampled were reared on pasture typical of the Mediterranean maquis without food supplementation. Only in the coldest winters was the ration supplemented with polyphyte hay (about 3 kg/head/day). In the summer, however, during the warmer weeks, the Podolica cows were moved to typical mountain pastures (about 1500 m above sea level).

As regards the herds of dairy cattle of other breeds (189 animals in total), 4 other farms with an intensive rearing system were sampled through the use of stationary automatic milking systems (AMSs). In the first farm, there were 54 Pezzata Rossa cows in lactation, which were fed a total mixed ration with dehydrated corn, straw, mixed hay, and concentrate once daily. In the second farm were 60 lactating cows of the Alpine brown breed, which were fed ad libitum once a day with a total mixed ration based on sorghum silage, hay, and concentrates. The herd conditions of the third and fourth farms were similar: 40 and 35 lactating Frisona cows fed corn silage for ad libitum consumption (4.5 kg of hay/d) in addition to concentrates.

Cows were milked twice a day in a milking parlor using automatic milking systems. In all farms, the milk collected came from both primiparous and pluriparous cows, and only healthy animals were included in the data analysis. All farms were located in the Calabria region (Italy), and all milk samples were collected between January 2022 and March 2023.

The bulk tank milk (BTM) samples (14 in total, representative of 120 Podolica cows and 189 cows of other breeds) were collected after the morning milking session.

After the milking session, the bulk milk was kept at 4 °C and with gentle shaking. A total of 1 L of milk was taken from the top of the tank using a clean, sanitized dipper after the milk was agitated for 5–10 min as previously described [19].

Samples were transported in a refrigerated state (≈0–4 °C) in a polystyrene insulated box filled with ice (water) to the “Centro Interdipartimentale di Servizi Veterinari” of Magna Graecia University of Catanzaro and analyzed within the same day.

### 2.2. FTIR Analysis

The protocol provided for heating of the samples in a water bath at 40 °C for 20 min and a short stirring period. Subsequently, the samples were analyzed with a MilkoScan FT+; in particular, 3–5 technical replicates were obtained for each sample. MIR spectra were acquired as transmittance values at 1060 points equally spaced in the mid-infrared region (wavenumber range from 925 to 5011 cm^−1^). Each measurement comprised 32 interferometer sub-scans, and FTIR spectra were recorded every millisecond. The nutritional composition was obtained for each sample taken (Table 1). The analysis of milk composition was performed according to the standards of the International Dairy Federation (FIL-IDF), and 4 technical replicates were acquired for each sample [4].

### 2.3. Statistical Analysis

The spectra recorded for each measurement were exported as CSV (Comma-Separated Values) files.

The exported CSV files were transformed into Excel files to create a single file as an excel matrix, which was analyzed through the multivariate discriminant analysis function of jmpSAS (version 14; SAS Institute Inc., Marlow, UK) statistical software. The spectra were used in their raw format with no pre-pretreatment or filtering and included all the pin number values in the analysis as an entire dataset. The protein, fat, lactose, and casein measured values were not included. Subsequently, jmpSAS multivariate discriminant analysis was used to build the models using a training set of analyzed samples that were randomly chosen from the entire sample set (training set). The remaining reads were used as a test set (analyzed in blind and to be recognized by the model). The jmp method was used for classification, which applied a multivariate model with discriminant analysis. Due to the high number of variables taken into consideration, the method applied was “Wide Linear”, which was validated through a blind analysis of excluded rows [20]. The evaluation of the 5 most influential absorbance regions/wavelength ranges was determined using the stepwise variable selection tool of jmpSAS, and the model was created by applying 5 steps to the classification.

## 3. Results

### 3.1. Pure Podolica Milk Recognition (Different Farms)

The analysis was performed on Podolica milk from three different farms, which was compared to milk from Frisona, Pezzata Rossa, and Bruna Alpina cattle from four different farms, thus allowing for the creation of a multivariate model based on a total of 68 reads of milk samples. In total, 38 reads were used as the training set and 30 reads as the test set. Among the tested samples, 34 reads were obtained from three different Podolica farms, and the remaining 34 were collected from four different farms rearing other bovine species (each farm was sampled at two different timepoints and analyzed four times, as described in the Materials and Methods section).

**Figure 1:**
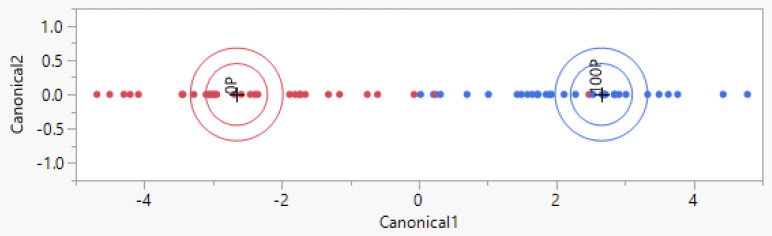
Representation of the biplot of the multivariate model built with jmpSAS used for the classification of Podolica milk and milk from three other breeds (0P stands for non-Podolica milk; 100P stands for 100% Podolica milk). The biplot axes are the first two canonical variables that define the two dimensions that provide maximum separation among the groups. The point corresponding to each multivariate mean is denoted by a plus (“+”) marker, while the first circle of each group represents the 95% confidence level ellipse. The second ellipse indicates a region in the space of the first two canonical variables that contains approximately 50% of the observations. If two groups differ significantly, the confidence ellipses tend not to intersect.

A representation of the multivariate model is visible in Figure 1. This model was created using the multivariate analytical tool of jmpSAS. The model was created using 38 reads for the training set, which was necessary for creation of the multivariate model. The same reads used to create the model were automatically re-analyzed (re-analysis over the training set) by the software, yielding a misclassification percentage of 0%. Moreover, the created model was used to analyze completely new samples to verify its power/reliability (blind analysis of the test set). This analysis yielded a misclassification percentage of 6.7% (0% in cases where a previous filtering step for outlier removal was added), as shown in the confusion matrix of Table 2.

### 3.2. Pure Podolica Milk Recognition (Same Farm)

**Figure 2:**
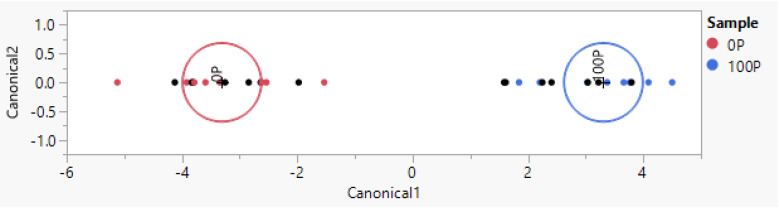
Representation of the multivariate model built with jmpSAS, which was used for the classification of Podolica milk and milk from Pezzata Rossa cattle reared by the same farm (0P stands for non-Podolica milk; 100P stands for 100% Podolica milk). The biplot axes are the first two canonical variables that define the two dimensions that provide maximum separation among the groups. The point corresponding to each multivariate mean is denoted by a plus (“+”) marker, while the first circle of each group represents the 95% confidence level ellipse. The second ellipse indicates a region in the space of the first two canonical variables that contains approximately 50% of the observations. If two groups differ significantly, the confidence ellipses tend not to intersect.

Podolica milk and Pezzata Rossa milk from the same farm, therefore with the same environmental conditions, could be distinguished with a 100% correct classification rate. The analysis was performed using 16 reads as the training set and 16 reads as a blind dataset. No errors were recorded in the recognition of both datasets, as visible in Figure 2 and as described in Table 3.

### 3.3. Recognition of Podolica Milk Contaminated with the Milk of Other Breeds (Different Farms)

**Figure 3:**
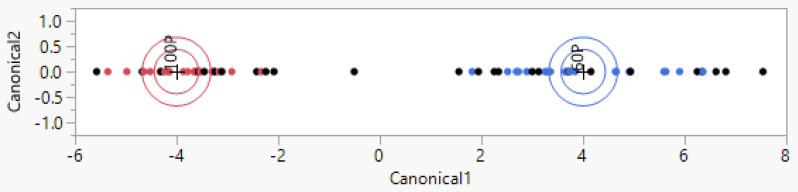
Representation of the multivariate model built with jmpSAS, which was used for the classification of Podolica milk and Podolica milk adulterated with 40% milk from other breeds from different farms.

It was possible to evaluate the detection of Podolica milk contaminated with 40% milk from different breeds. In this case, 40 samples of different mixtures were used as the training set to recognize 28 blind samples. The misclassification percentage among the training set and the test set was 0% in both cases. The model is visible in Figure 3, and Table 4 briefly describes the results. Another test was performed to try to detect Podolica milk adulterated with 20% of other bovine breeds’ milk, which yielded a misclassification percentage of 26.7% for the test set and sensitivity and specificity of 73.3% for the detection of the 20% adulteration condition.

### 3.4. Recognition of Podolica Milk Contaminated with Milk from Other Breeds (Same Farm)

**Figure 4:**
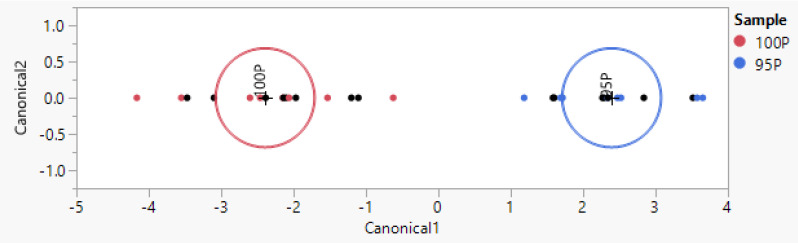
Representation of the multivariate model built with jmpSAS, which was used for the classification of Podolica milk and Podolica milk adulterated with 5% milk from Pezzata Rossa cattle from the same farm.

Removing the environmental variability and building a model with milk from a different breed (Pezzata Rossa) growing in the same environment, it is possible to detect up to 5% of contamination. In this case, i.e., the recognition of different breeds from the same farm, the model was trained with 16 known samples and tested with 16 unknown samples from different sample collections and different mixture replicates. The test yielded 6.2% misclassification in the training set and 100% accuracy in the test set (Figure 4 and Table 5). For the completeness of the test, contamination concentrations of 20% and 10% were tested, yielding a 100% correct classification rate in the training set and test set. Only results detecting the lowest of the tested contamination concentrations were reported.

## 4. Discussion

The Food and Agriculture Organization of the United Nations (FAO) has estimated the extinction of 300 out of 6000 breeds of all farm animal species. The Podolica breed is, among cows, seriously endangered in various European areas [2]. However, its associated high-quality dairy products, such as milk and Caciocavallo cheese, are generating growing interest in the Podolica breed. Podolica milk is higher in protein, fat, and somatic cell content than Friesian milk. Furthermore, Podolica cheese has a higher content of C6:0 (caprylic acid) and C14:0 (myristic acid) fatty acids, as well as stearic and linolenic acid, while total palmitic, palmitoleic, oleic, and linoleic fatty acids are less represented [21]. Due to its valuable nutritional properties, Podolica milk and its derived products have a higher economic value, and there is therefore a necessity for a rapid and cheap method for the recognition of this product.

Fourier Transform Infrared Spectroscopy has often been used for the differentiation of milk from different species [16,22], including the detection of milk composition changes according to housing modification [14], the detection of subclinical ketosis [23], and, coupled with multivariate analysis, the differentiation of goat milk breeds [24]. However, further studies are needed to establish its role in the differentiation of milk from bovine breeds.

This would provide a cheap method indispensable in routine milk analysis and become widely used as a rapid and comprehensive means of assessing various quality parameters and detecting adulterations. The analytical speed and cost-effectiveness would make this technology a good candidate for the purposes herein proposed. Moreover, its use in routine analysis would make it possible to collect a high number of datasets that could be used to train the multivariate models that, in turn, would become better and better as the dimensions of the datasets grow.

As in the results here described, FTIR coupled with machine learning offered the possibility of rapidly classifying milk samples from different bovine breeds.

The first multivariate discriminant model was created to automatically differentiate Podolica milk samples from milk from other bovine species (Figure 1). The analysis on the test set yielded a misclassification percentage of 6.7% (Table 2). Among the 15 non-Podolica samples in the test set, 2 were misclassified and recognized as Podolica milk even though the milk was from other species (86.7% sensitivity for the detection of non-Podolica milk). Considering this positive result in the detection of non-Podolica milk, attempts to correctly classify different mixtures were undertaken, leading to considerable results when the mixture of Podolica milk with non-Podolica milk reached 40%. In Figure 3 and Table 4, the multivariate model and the respective confusion matrices yielded are shown. Among 14 Podolica samples mixed with 40% non-Podolica milk, none were misclassified as 100% Podolica milk (100% sensitivity). Podolica milk adulterated with 20% of other bovine breeds’ milk yielded a misclassification percentage of 26.7% for the test set and sensitivity and specificity of 73.3% for the detection of the 20% adulteration condition (see Supplementary Figure S1).

The correct classification rate improved to 100% when the same method was applied to the recognition of Podolica and Pezzata Rossa milk reared by the same farm (Figure 2, Table 3). Considering this positive result, it was decided that attempts should be made to detect the contamination of Pezzata Rossa milk when added to Podolica milk from the same farm. The measured concentrations of 20% and 10% yielded a specificity and sensitivity of 100% in the training set and in the test set, and it was possible to detect as little as 5% contamination with a 100% correct classification rate in both the training and the test set (100% sensitivity and 100% specificity, see Table 5).

The method here described yielded higher accuracy values when applied to the discrimination of milk from different breeds belonging to the same farm. One reason for this phenomenon might be linked to the elimination of the environmental variable. In this case, the bulk milk samples were collected separately from the two different bovine species reared at the same geographical location. Moreover, the detection of Podolica milk among the other three different bovine breeds was made more difficult by the enhanced variability of the control group, which was composed of three different breeds rather than one.

These results are in line with the fact that cattle breeds and their related genetic traits can influence milk composition [25,26], even if not much is known about which genes are responsible for FTIR milk spectral profile shaping [27]. Some studies about this topic recently have demonstrated an association between FTIR milk spectra and the quantitative trait loci of two different Danish dairy cattle breeds [27]. Moreover, FTIR milk spectra have been linked to genes associated with milk composition, such as diacylglycerol O-acyltransferase 1 (DGAT1) and β-lactoglobulin (PAEP) [28].

As for the Jersey breed, the wavenumbers that provide interaction with the C-H group correspond to genes responsible for the synthesis of fatty acids, while for the wavenumbers that interact with the −OH group, an association was found with the genes involved in the synthesis of α-lactalbumin [27].

In addition, FTIR spectroscopy was used to perform genome-wide association analyses for fatty acids present in the milk of 1811 Norwegian Red cattle. The study conducted is very important as bovine milk has a high content of saturated fatty acids (FAs), especially palmitic acid (C16:0), which is particularly harmful to the cardiovascular system. If palmitic acid were replaced with unsaturated FAs, such as oleic acid (C18:1cis-9), bovine milk would be healthier. Therefore, knowing the sequence of the whole genome, FTIR allowed for the identification of genetic variants that have opposite effects on the levels of C16:0 and C18:1cis-9, defining the transcription profile and the protein content of the candidate genes. In particular, in association with BTA 11, the PAEP (progestagen-associated endometrial protein) gene has been identified, which is translated into the milk protein β-lactoglobulin. The genetic variants identified allowed for definition of a favorable haplotype associated with low levels of C16:0 and high levels of C18:1cis-9, as well as lower expression of PAEP and lower β-lactoglobulin content. Lower content of this protein in milk results in a better yield during cheese production. Therefore, FTIR made it possible to identify genetic variants that would allow farmers both to produce milk that is healthier in terms of FA content and to improve the properties of dairy products [29].

Breed-associated differences in FTIR milk spectra and genetic traits could provide the basis for future research about associations between FTIR spectroscopy and not only adulterations and traceability, but also the possible detection of links between the FTIR wavelengths and eventually related genetic traits.

In our case, classification using stepwise variable selection (using all samples) considered the pin numbers 265, 283, 396, 401, and 426 as the most relevant for the classification of the entire dataset of the Podolica breed versus the other three different breeds.

The relationship between wavenumbers and pin numbers is as follows: wavenumber cm^−1^ = 3.858 × pin number [30].

**Figure 5:**
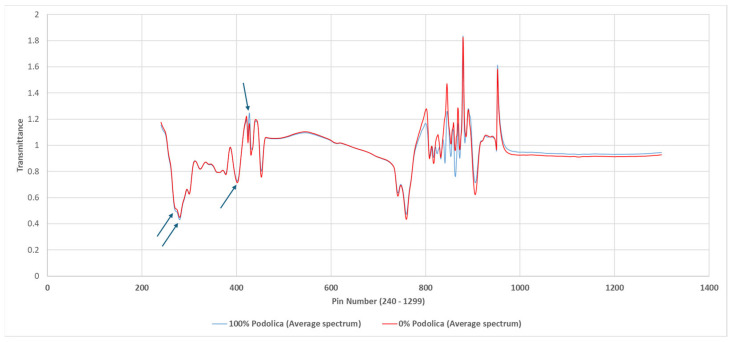
Representation of the average spectra of Podolica milk (blue) and non-Podolica milk (red). The blue arrows represent the spectral regions with the major variability.

In Figure 5, the average spectrum of the two different most extreme groups (0% Podolica and 100% Podolica) is shown, with the most variable regions (according to the variability of the pin numbers) highlighted by the blue arrows.

It is now known in the literature that the specific absorbance bands/regions of the mid-infrared spectrum provide us with information on the composition of milk. In our study, the first absorption region relevant to the classification of Podolica milk versus other cow breeds’ milk was the one at 1022 and 1092 cm^−1^, which corresponds to lactose [31]. Subsequently, the absorbing regions at 1528, 1547, and 1644 cm^−1^ contained the peaks typical of amide I (C-O) and amide II (N–H) and are therefore associated with proteins [32].

This study presents models that were obtained using the samples available in this regional environment. However, more robust, universal, and accurate models might be obtained by enlarging the number of samples used in the training sets. This task is achievable considering that FTIR is one of the most commonly used techniques for routine milk analysis. Thus, improving the dimension and heterogeneity of the test sets could be feasible within a reasonable amount of time and with moderate financial effort.

## 5. Conclusions

The results herein described demonstrate that FTIR spectral profiling coupled with machine learning could be capable of detecting milk other than that produced by the Podolica breed. It is interesting to underline that, when eliminating the environmental variable, the method applied was more accurate and capable of detecting lower amounts of non-Podolica milk as a contaminant.

More robust and accurate models might be obtained by enlarging the number of samples in the training sets. This task is achievable considering that FTIR is one of the most commonly used techniques for routine milk analysis. Thus, improving the dimension and heterogeneity of the test sets could be feasible within a reasonable amount of time and with moderate financial effort.

Considering that FTIR spectral profiling is routinely used for milk analysis because of its speed and cost-effectiveness, this computational approach might add analytical power to this technique.

## Figures and Tables

**Table 1:** Mean ± standard deviation of the fat, protein, lactose, and casein content of the milk samples observed for the two experimental times.

	Podolica	Frisona	Pezzata Rossa	Bruna Alpina
Fat (g/100 g)	2.92 ± 0.34	3.64 ± 0.39	3.83 ± 0.25	4.04 ± 0.24
Protein (g/100 g)	3.34 ± 0.21	3.07 ± 0.14	3.29 ± 0.05	3.35 ± 0.25
Lactose (g/100 g)	4.93 ± 0.10	4.75 ± 0.11	4.92 ± 0.19	4.73 ± 0.14
Casein (g/100 g)	2.71 ± 0.09	2.54 ± 0.26	2.68 ± 0.13	2.64 ± 0.17

**Table 2:** Confusion matrices reporting the recognition of Podolica milk and non-Podolica milk samples.

			Test Set		
Actual	Predicted Count		Actual	Predicted Count	
Class	0% Podolica	100% Podolica	Class	0% Podolica	100% Podolica
0% Podolica	19	0	0% Podolica	13	2
100% Podolica	0	19	100% Podolica	0	15
Sensitivity for 0% Podolica		100%	Sensitivity for 0% Podolica		86.7%
Specificity for 0% Podolica		100%	Specificity for 0% Podolica		100%

**Table 3:** Confusion matrices of the recognition of Podolica milk and Pezzata Rossa milk from cattle reared by the same farm.

			Test Set		
Actual	Predicted Count		Actual	Predicted Count	
Class	0% Podolica	100% Podolica	Class	0% Podolica	100% Podolica
0% Podolica	8	0	0% Podolica	8	0
100% Podolica	0	8	100% Podolica	0	8
Sensitivity for 0% Podolica		100%	Sensitivity for 0% Podolica		100%
Specificity for 0% Podolica		100%	Specificity for 0% Podolica		100%

**Table 4:** Confusion matrices showing the results of the application of the model for the detection of Podolica milk adulterated with 40% milk from other breeds.

			Test Set		
Actual	Predicted Count		Actual	Predicted Count	
Class	100% Podolica	60% Podolica	Class	100% Podolica	60% Podolica
100% Podolica	20	0	100% Podolica	14	0
60% Podolica	0	20	60% Podolica	0	14
Sensitivity for 60% Podolica		100%	Sensitivity for 60% Podolica		100%
Specificity for 60% Podolica		100%	Specificity for 60% Podolica		100%

**Table 5:** Confusion matrices showing the results of the application of the model for the detection of Podolica milk adulterated with 5% Pezzata Rossa milk from the same farm.

			Test Set		
Actual	Predicted Count		Actual	Predicted Count	
Class	100% Podolica	95% Podolica	Class	100% Podolica	95% Podolica
100% Podolica	8	0	100% Podolica	8	0
95% Podolica	0	8	95% Podolica	0	8
Sensitivity for 95% Podolica		100%	Sensitivity for 95% Podolica		100%
Specificity for 95% Podolica		100%	Specificity for 95% Podolica		100%

## Data Availability

The data are contained within the article.

## Supplementary Materials

The following supporting information can be downloaded at: https://www.mdpi.com/article/10.3390/ani14091271/s1, Figure S1. a. Representation of the multivariate model built with jmpSAS, which was used for the classification of Podolica milk and Podolica milk adulterated with 20% milk from other breeds from different farms. b. Confusion matrices showing the results of the application of the model for the detection of Podolica milk adulterated with 20% milk from other breeds.
