# Supplementary figures and images for: Spectral Profiling (Fourier Transform Infrared Spectroscopy) and Machine Learning for the Recognition of Milk from Different Bovine Breeds
Source: Animals (Basel). 2024 Apr 24;14(9):1271. doi: 10.3390/ani14091271 (PMC11083570; doi:10.3390/ani14091271)

**a**

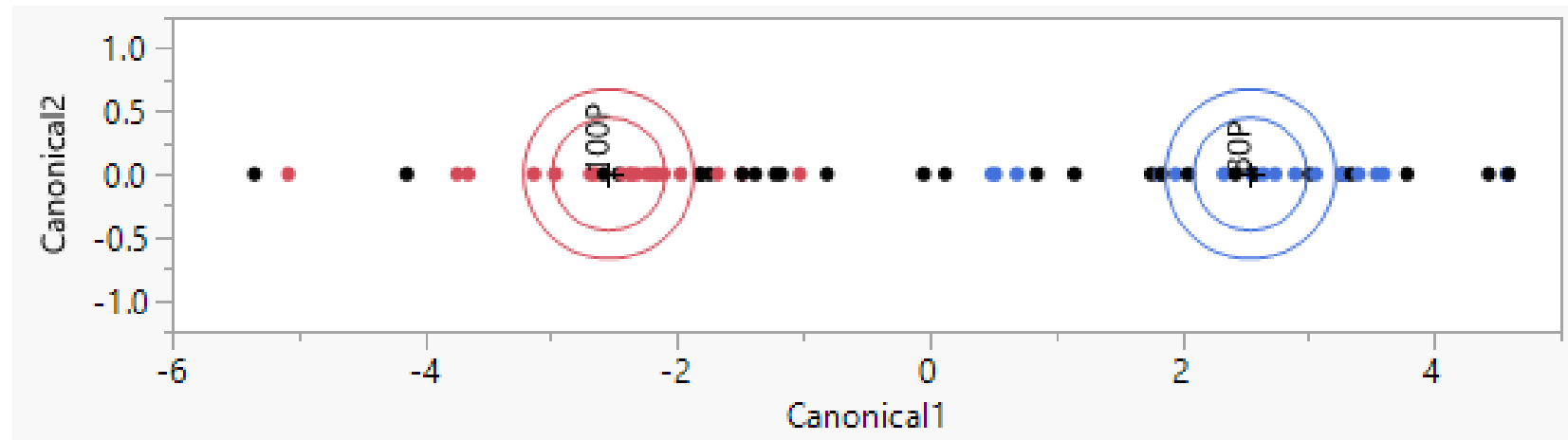

**b**

| Training |                 |     | Excluded |                 |     |
|----------|-----------------|-----|----------|-----------------|-----|
| Actual   | Predicted Count |     | Actual   | Predicted Count |     |
| Sample   | 100P            | 80P | Sample   | 100P            | 80P |
| 100P     | 19              | 0   | 100P     | 11              | 4   |
| 80P      | 0               | 19  | 80P      | 4               | 11  |

Supplement: Supplementary file 1 [file animals-14-01271-s001.zip › animals-2918657-supplementary.pdf]
